# The Interplay of Cx26, Cx32, Cx37, Cx40, Cx43, Cx45, and Panx1 in Inner-Ear Development of Yotari (dab1−/−) Mice and Humans

**DOI:** 10.3390/biomedicines10030589

**Published:** 2022-03-03

**Authors:** Josip Lesko, Pejana Rastović, Josip Mišković, Violeta Šoljić, Vlatka Paštar, Zdenka Zovko, Natalija Filipović, Yu Katsuyama, Mirna Saraga-Babić, Katarina Vukojević

**Affiliations:** 1 Department of Anatomy, School of Medicine, University of Mostar, 88000 Mostar, Bosnia and Herzegovina; josip.lesko@mef.sum.ba (J.L.); pejana_rastovic@mef.sum.ba (P.R.); josip.miskovic@mef.sum.ba (J.M.); zdenka.zovko@mef.sum.ba (Z.Z.); 2 Department of Histology and Embryology, School of Medicine, University of Mostar, 88000 Mostar, Bosnia and Herzegovina; violeta.soljic@mef.sum.ba; 3 Faculty of Health Studies, University of Mostar, 88000 Mostar, Bosnia and Herzegovina; 4 Mediterranean Institute for Life Sciences (MedILS), 21000 Split, Croatia; vlatka.pastar@medils.hr; 5 Department of Anatomy, Histology and Embryology, School of Medicine, University of Split, Šoltanska 2, 21000 Split, Croatia; natalija.filipovic@mefst.hr (N.F.); msb@mefst.hr (M.S.-B.); 6 Department of Anatomy, Shiga University of Medical Science, Otsu 520-2192, Japan; kats@belle.shiga-med.ac.jp

**Keywords:** gap junction, inner-ear development, Cx26, Cx32, Cx37, Cx40, Cx43, Cx45, Panx1, yotari

## Abstract

We investigated DAB1-protein deficiency in the inner-ear development of *yotari* in comparison to humans and wild-type (wt) mice by immunofluorescence for the expression of connexins (Cxs) and the pannexin Panx1. The spatial and temporal dynamics of Cx26, Cx32, Cx37, Cx40, Cx43, Cx45, and Panx1 were determined in the sixth and eighth weeks of human development and at the corresponding mouse embryonic E13.5 and E15.5, in order to examine gap junction intercellular communication (GJIC) and hemichannel formation. The quantification of the area percentage covered by positive signal was performed for the epithelium and mesenchyme of the cochlear and semicircular ducts and is expressed as the mean ± SD. The data were analysed by one-way ANOVA. Almost all of the examined Cxs were significantly decreased in the cochlear and semicircular ducts of yotari compared to wt and humans, except for Cx32, which was significantly higher in *yotari*. Cx40 dominated in human inner-ear development, while yotari and wt had decreased expression. The Panx1 expression in yotari was significantly lower than that in the wt and human inner ear, except at E13.5 in the mesenchyme of the wt and epithelium and mesenchyme of humans. Our results emphasize the relevance of GJIC during the development of vestibular and cochlear functions, where they can serve as potential therapeutic targets in inner-ear impairments.

## 1. Introduction

The inner ear is made up of the vestibular and cochlear segments, which develop from the otic vesicle, a specialized epithelial invagination of ectodermal origin that gives rise to specific sensory structures: the macula sacculi, macula utriculi, cristae ampullares, and cochlear duct [1]. The progressive development of the human inner ear begins around the fourth week of gestation from the otic placodes towards the otic vesicle. The otic vesicle is divided into the ventral and dorsal components, from which the vestibular (semicircular canals and associated cristae, saccule, and utricle) and cochlear segments (cochlea) emerge. These segments provide balance and spatial orientation through the vestibular organ and hearing perception through the cochlea [2,3]. At the end of the eighth week of gestation, the sensory organs of the inner ear obtain their final contours [4]. In various organ systems of mice as well as of humans, at the level of the cell membrane, special proteins called connexins (Cxs) build larger units of the cell membrane, called connexons, each consisting of six connexin subunits. Connexons are called hemichannels because they connect intracellular and extracellular spaces; they open under different influences and allow substances to flow from one space to another. Two hemichannels between neighbouring cells form a gap junction, which allows intercellular communication by facilitating the diffusion of ions and small metabolites important for metabolic homoeostasis [5]. The majority of the cells in the inner ear are extensively coupled via gap junctions, thus implying a key role for gap junctional communication in the inner-ear network [6]. Previous studies suggest that humans have 21 Cxs, while mice have 20 types of Cxs, 19 of which are matching pairs [7]. Connexins are thought to play an important role in the development of the ear, for the very structure of the cochlea, as well as for the endocochlear potential [8]. Communication between cells is particularly important in tissues that have poor and complex circulation, which includes the circulation of the inner ear [9]. The main focus in previous studies was on the connexins Cx26 and Cx30, which were found to be present in the cochlea, mainly in non-sensory cells [9] The importance of connexins for normal cochlear function is shown by genetic mutations, which, in the case of genes encoding Cx26, cause almost half of hereditary prelingual deafness [10]. In addition, recent studies showed that mutations in genes encoding other connexins, such as Cx29, Cx30, Cx31, and Cx32, have an effect on cochlear function impairment and deafness [11]. Other connexins were also found in the structures of the inner ear, both during embryonic development and in the postnatal period. Those were primarily Cx43 and Cx45, which were found during embryonic development in epithelial tissue and connective tissue components [12]. Later on, during the postnatal period, Cx45 was mainly found in the vascular structures of the inner ear [13]. Disordered Cx45 expression could be associated with vascular abnormalities in the inner ear, disturbances in normal intercellular functioning, and, consequently, a loss of function, such as impaired hearing [13]. Contrary to connexins, pannexins do not form gap junctions, and they provide an intracellular–extracellular conduit [8,14]. There are three isoforms of pannexins—Panx1, Panx2, and Panx3—and all of them were isolated in cochlear structures, but Panx1 was predominantly so [15]. Pannexin 1 can serve as an alternative moderator of intercellular signalling and the principal conduit of ATP release from dying cells [10,16]. Studies have shown that Panx1 deficiency can cause hearing impairment, which might imply a role for it in hearing acquisition and auditory function [17].

Yotari mice are autosomal recessive mutant mice, produced by the mutation of the disabled homolog 1 (Dab1) gene, and are recognized by their unstable gait, tremor, and early deaths at around P21 [12]. Therefore, the signalling pathway in the brain reelin–disabled-1 (DAB1) is very important for the normal development of brain structures. Reelin is an extracellular glycoprotein of the cell membrane, whose activation stimulates further signalling processes within the cell itself, including activating the DAB1 protein. When activated, DAB1 stimulates cellular processes that are essential for cell developmental activities. This canonical signalling pathway is essential for the development of neurons and their migration, and, later on, for the proper functioning of the nervous system [18]. Investigations in this field indicated that dysfunction of the components of this signalling pathway can cause various outbursts in brain development and, thus, their later clinical manifestations [19]. The inactivation of reelin and DAB1 in mice appears to have similar clinical manifestations, such as unstable gait, tremor, and early death [20].

Since the DAB1 protein is important in the developmental processes of the nervous system, in this study, we wanted to investigate the impact of DAB1-protein deficiency on the development of vestibular and cochlear functions, followed through the expression of connexins and pannexins. In this study, the distinct distribution patterns of Cxs and Panx1 in the inner ears of human conceptuses, and wild-type and yotari mice were demonstrated, indicating their relevance in early inner-ear development.

## 2. Materials and Methods

### 2.1. Animal Model, Human Samples, and Tissue Processing

In this study, Yotari (Dab1 dab1−/−) C57BL/6N mice that were previously described [1,2] were used as Dab1-null conventional mutants. The mice were housed in polycarbonate cages in a temperature-controlled (23 ± 2 °C) room with a 12 h dark/light cycle and unlimited access to food and water. Gravid mice were anesthetized with pentobarbital, followed by transcardial perfusion using phosphate buffer saline (PBS) with a pH of 7.2 and 4% paraformaldehyde in 0.1 M PBS, and embryos (E) were sacrificed on E13.5 and E15.5 embryonic days. The embryos were separately fixed in 4% paraformaldehyde in 0.1 M PBS overnight for histological analyses: haematoxylin–eosin (H&E) and immunofluorescence (IF) staining. For genotyping, the following PCR primers were used: yotari—GCCCTTCAGCATCACCATGCT and CAGTGAGTACATATTGTGTGAGTTCC; wild type—GCCCTTCAGCATCACCATGCT and CCTTGTTTCTTTGCTTTAAGGCTGT [20,21].

Eight human samples of embryonic and foetal heads containing inner ears were obtained from the archival collection of histological sections stored at the Department of Anatomy, Histology and Embryology, School of Medicine, University of Split. The conceptuses were of the 6th and 8th developmental week (according to Carnegie stage measurements), to correspond to mice at E13.5. and E15.5, respectively. The human samples (spontaneous abortions or tubar pregnancies) were without any morphological signs of abnormality or macerations and were collected after obtaining the permission of the Ethical and Drug Committee of the University Hospital of Split (class: 003-08/16-03/0001, registry number: 2181-198-03-04-16-0024, 20 May 2016) in accordance with the Helsinki Declaration.

### 2.2. Immunofluorescence

After fixation and dehydration, tissues were embedded in paraffin blocks, sliced into 5 µm-thick consecutive sections, and mounted on microscopic slides. Proper tissue preservation was verified with the H&E staining of every 10th section. Following xylol deparaffinization and ethanol/distilled water rehydration, for antigen retrieval, tissue samples were heated in a sodium citrate buffer (pH6) for 17 min at 95 °C, cooled down to room temperature, and washed with PBS. Protein blocking buffer (ab64226, Abcam, Cambridge, UK), applied for 30 min, was used to prevent nonspecific binding, followed by the samples’ incubation with primary antibodies (Table 1) overnight in a humidity chamber. The next day, the samples were rinsed with PBS and incubated with suitable secondary antibodies (Table 1) for one hour. Afterward, the slides were rinsed with PBS again and the nuclei were stained with 40,6-diamidino2-phenylindole (DAPI), rinsed with distilled water, and mounted on microscopic slides (Immuno-Mount, Thermo Shandon Cheshire, UK). A light microscope (for H&E tissue sections) and fluorescence microscope (Olympus BX51, Tokyo, Japan) with a Nikon DS-Ri1 camera (Nikon Corporation, Tokyo, Japan) were used to obtain images.

### 2.3. Statistical Analysis

For the statistical analyses of all the obtained results, the GraphPad Software was used (GraphPad Prism v.8, La Jolla, CA, USA), with a confidence level of *p* < 0.05 considered as statistically significant. The ImageJ software (National Institutes of Health, Bethesda, MD, USA) and Adobe Photoshop (Adobe, San Jose, CA, USA) were used for image analysis. Ten non-overlapping representative visual fields of the mouse embryonic inner ear were taken using 100× magnification applied with immersion oil with the same camera settings (Carl Roth, Karlsruhe, Germany). Green staining was interpreted as positivity for Cx26, Cx32, Cx37, Cx40, Cx43, Cx45, and Panx1 immunoexpression. The subtraction of the median filter and colour thresholding to calculate the section percentage area covered by the positive signal was used to obtain a quantitative estimation of the immunoreactivity. For the comparison of the immunoexpression to determine significant differences between groups, a one-way ANOVA test followed by a post hoc Tukey’s test was used. The data are summarized as mean ± SD. Three investigators analysed the images independently, while three to four tissue samples were used per group in each replicated experiment (*n* ≥ 3).

## 3. Results

### 3.1. Mouse and Human Inner-Ear Development

**Figure 1:**
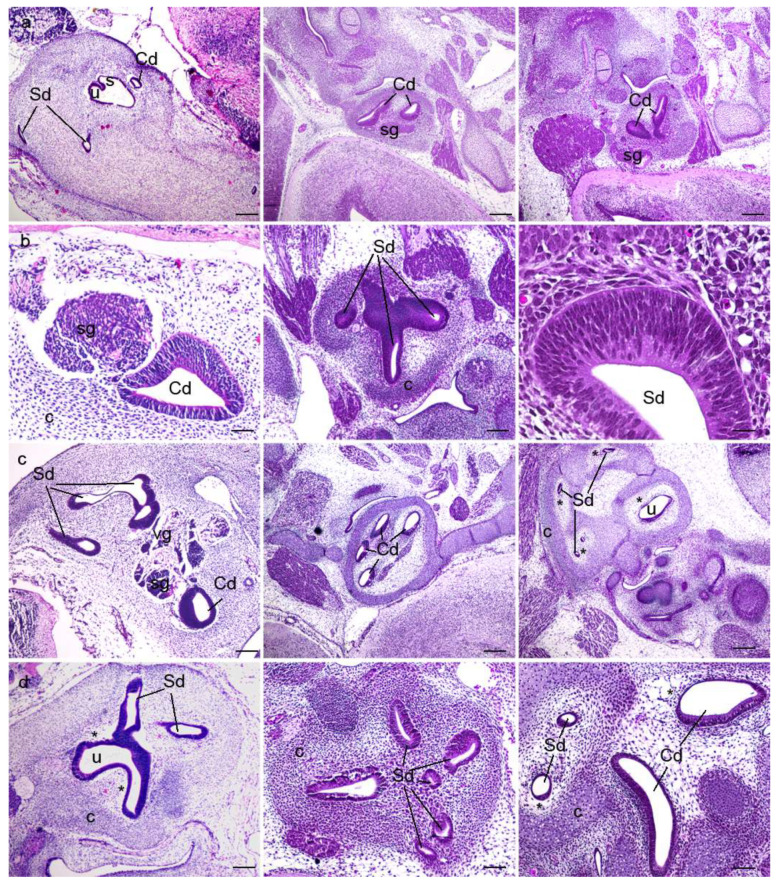
Haematoxylin–eosin (HE) staining of the human, and wild-type (wt) and *yotari* mouse inner ears. HE staining of the human (first column), wt (second column), and *yotari* (third column) inner ears in the 6th (**a**,**b**) and 8th (**c**,**d**) weeks of development (6w/8w)/the embryonic day 13.5 (**a**,**b**) and E15.5 (**c**,**d**) (E13.5 and E15.5). Semicircular ducts (Sd), cochlear duct (Cd), saccule (s), utricle (u), vestibular ganglion (vg), spiral ganglion (sg), the cartilaginous shell (c), and the perilymphatic spaces (*). The scale bar for panels in the first (**a**) and third (**c**) rows equals 200 μm; that for panels in the second row (**b**) equals 40 μm, 100 μm, and 25 μm, respectively; that for panels in the fourth (**d**) row equals 100 μm.

The morphological aspects of the human, and wild-type and yotari mouse inner ears in the sixth and eighth weeks of human development and at the corresponding stages of mouse development, E13.5 and E15.5, were analysed using haematoxylin–eosin staining (Figure 1). During inner-ear development, vestibular and cochlear regions differentiated from the otocyst in the dorsal and ventral aspect, respectively. In the sixth week of development, semicircular canals and the utricle had already developed from the vestibular portion, whereas the cochlear duct and the saccule had already developed from the cochlear region (Figure 1a). During development, the epithelium of the vestibulocochlear region induces the adjacent mesenchyme to condense and form the cartilaginous shell. Parts of the cartilaginous shell adjacent to epithelium undergo vacuolization to form the perilymphatic spaces (Figure 1). Although all the components are the same in both human and mouse inner-ear development, we observed a markedly thicker and more cellular cochlear duct epithelium in humans than in mice in the eighth week of development and E15.5, respectively (Figure 1c). During the eighth week of development, the membranous labyrinth enlarges within its cartilaginous shell, which serves as a backbone of the bony labyrinth and otic capsule in the later developmental stages.

### 3.2. Connexins (Cxs) and Pannexin 1 (Panx1) Expression

The immunoexpression of Cxs (Cx26, Cx32, Cx37, Cx40, Cx43, and Cx45) and Panx1 were investigated in the human, and wild-type and *yotari* mouse inner ears in the sixth and eighth weeks of human development and at the corresponding mouse stages, E13.5 and E15.5, in order to examine the gap junction intercellular communication (GJIC) and the hemichannel formation.

**Figure 2:**
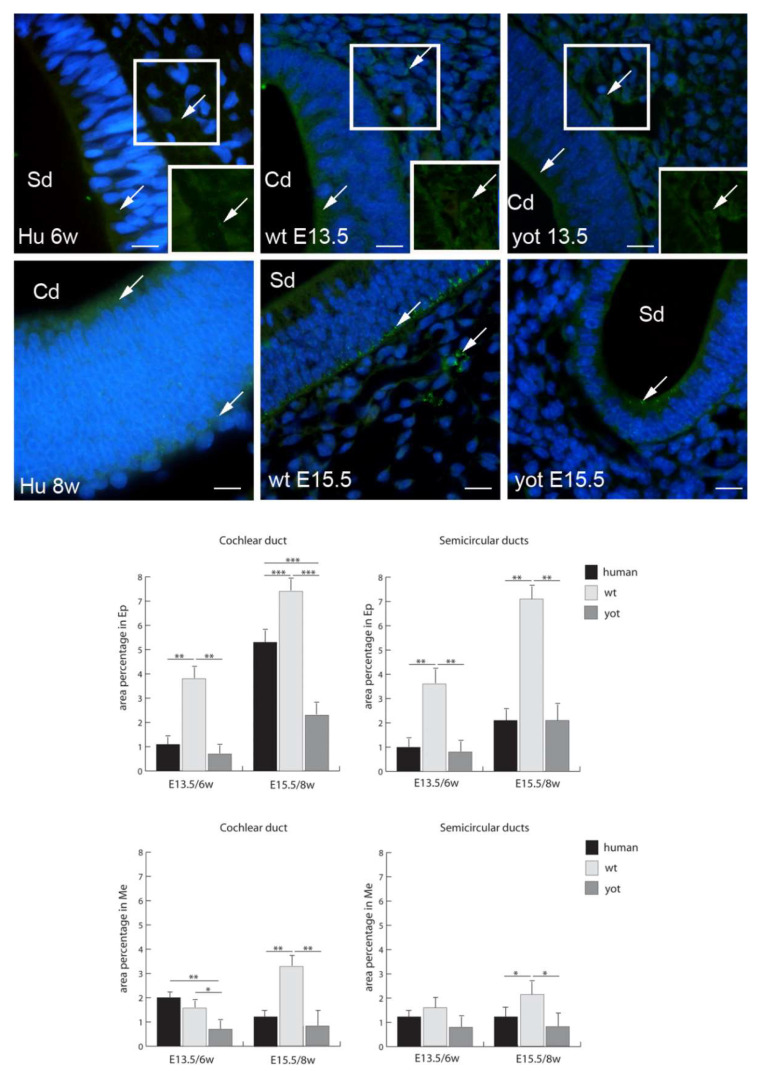
Immunofluorescence staining of connexin Cx26 (arrows) in the human (Hu), and wild-type (wt) and *yotari* (yot) mouse inner ears in the 6th and 8th weeks of human development and at corresponding E13.5 and E15.5, merged with DAPI (blue nuclear stain). Scale bar is 10 µm, for all images. The panel with graphs represents the area percentages of connexin Cx26 in the human, and wild-type and yotari mouse inner ears in the 6th and 8th weeks of human development and at corresponding E13.5 and E15.5 in epithelium and mesenchyme of cochlear duct and semicircular ducts. Sd (semicircular ducts), Cd (cochlear duct), Ep (epithelium), and Me (mesenchyme). Data are presented as the mean ± SD (vertical line). Significant differences are indicated by * *p* < 0.05, ** *p* < 0.01, and *** *p* < 0.001. One-way ANOVA followed by Tukey’s multiple-comparisons test.

Cx26 had the highest expression in the epithelium of both the cochlear duct and semicircular ducts in wild-type mice at E15.5, while the lowest expression was in the epithelium and mesenchyme of the cochlear duct at E13.5 (Figure 2). Through the human developmental weeks six and eight as well as through the embryonic stages E13.5 and E15.5 in wt and *yotari* mice, there was a strong increase in Cx26 expression, except for the mesenchyme of the cochlear duct in the human inner ear in the eighth week of development, where we observed a decrease from the sixth week to the eighth week of development (Figure 2). The expression of Cx26 in *yotari* mice was always significantly lower in all the structures and stages in comparison to wt mice. Additionally, the Cx26 expression in the human inner-ear developmental stages was significantly lower than in the corresponding wt mouse stages, except in the mesenchyme of the cochlear duct in the sixth week of development (Figure 2).

**Figure 3:**
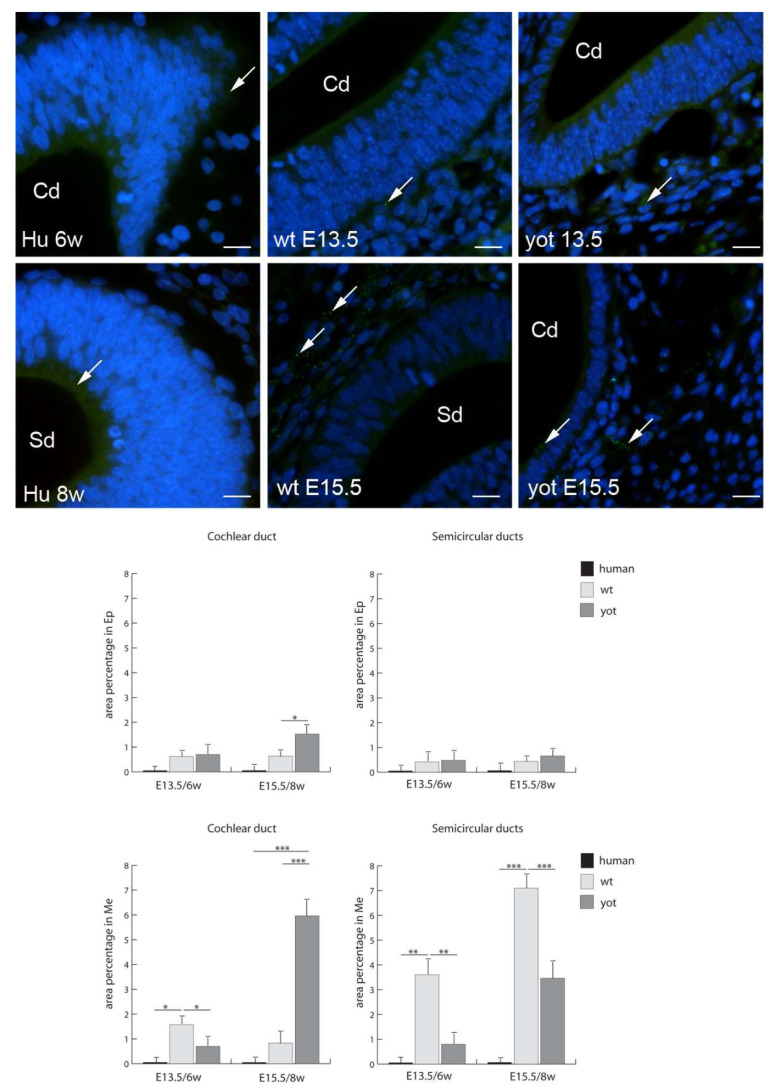
Immunofluorescence staining of connexin Cx32 (arrows) in the human (Hu), and wild-type (wt) and *yotari* (yot) mouse inner ears in the 6th and 8th weeks of human development and at corresponding E13.5 and E15.5, merged with DAPI (blue nuclear stain). Scale bar is 10 µm, for all images. The panel with graphs represents the area percentages of connexin Cx32 in the human, and wild-type and yotari mouse inner ears in the 6th and 8th weeks of human development and at corresponding E13.5 and E15.5 in epithelium and mesenchyme of cochlear duct and semicircular ducts. Sd (semicircular ducts), Cd (cochlear duct), Ep (epithelium), and Me (mesenchyme). Data are presented as the mean ± SD (vertical line). Significant differences are indicated by * *p* < 0.05, ** *p* < 0.01, and *** *p* < 0.001. One-way ANOVA followed by Tukey’s multiple-comparisons test.

Cx32 had the highest expression in the mesenchyme of the semicircular ducts of wt mice and cochlear ducts of *yotari* mice at E15.5 (Figure 3). While insignificant expression of Cx32 was observed for all the human stages, both wt and yotari had very low Cx32 expression in the epithelium of the cochlear duct and semicircular ducts. At E15.5, in the cochlear duct, *yotari* mice had significantly higher expression of Cx32 than wt mice in both the epithelium and mesenchyme. Contrary to that, in the mesenchyme of the cochlear duct (E13.5) and semicircular ducts (E13.5. and E15.5), Cx32 expression was significantly lower than in wt mice (Figure 3).

**Figure 4:**
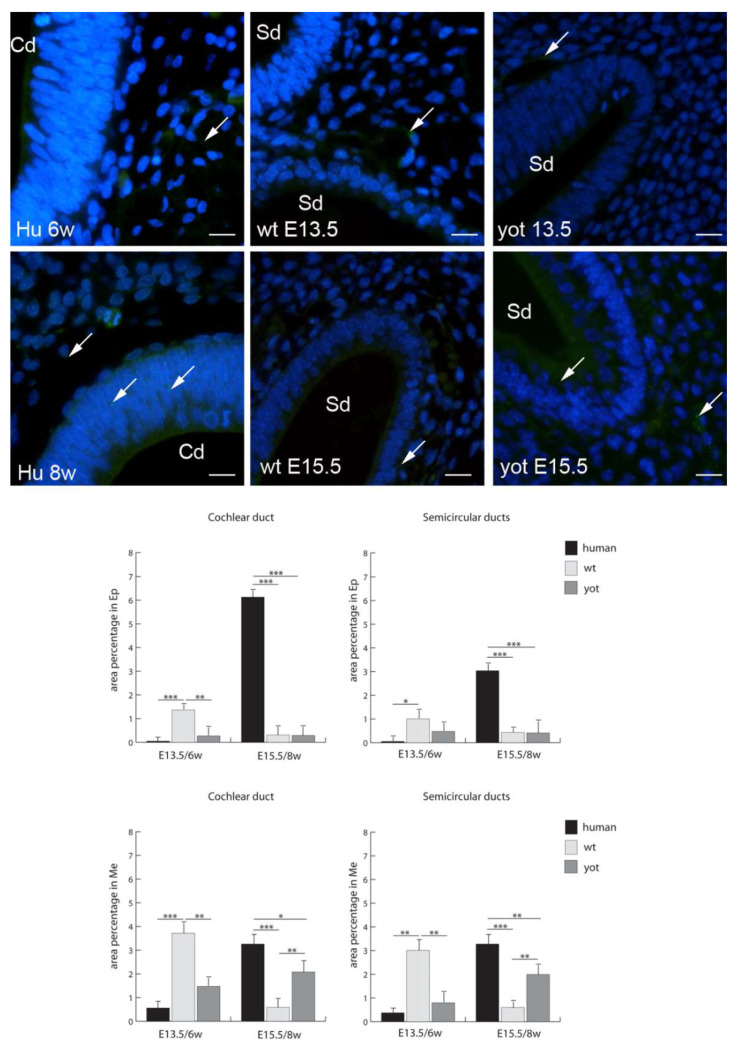
Immunofluorescence staining of connexin Cx37 (arrows) in the human (Hu), and wild-type (wt) and *yotari* (yot) mouse inner ears in the 6th and 8th weeks of human development and at corresponding E13.5 and E15.5, merged with DAPI (blue nuclear stain). Scale bar is 10 µm, for all images. The panel with graphs represents the area percentages of connexin Cx37 in the human, and wild-type and yotari mouse inner ears at 6th and 8th weeks of human development and at corresponding E13.5 and E15.5 in epithelium and mesenchyme of cochlear duct and semicircular ducts. Sd (semicircular ducts), Cd (cochlear duct), Ep (epithelium), and Me (mesenchyme). Data are presented as the mean ± SD (vertical line). Significant differences are indicated by * *p* < 0.05, ** *p* < 0.01, and *** *p* < 0.001. One-way ANOVA followed by Tukey’s multiple-comparisons test.

Cx37 had the highest expression in the epithelium of the cochlear duct in the human inner ear in the eighth week of development and in the mesenchyme of the cochlear duct in wild-type mice at E13.5. The lowest expression of Cx37 was in the epithelium of the cochlear duct and semicircular ducts in the sixth week of human inner-ear development (Figure 4). From the sixth to the eighth week of human inner-ear development, there was a strong increase in Cx37 expression, while wt mice in the corresponding stages had a strong decrease in Cx37 expression, especially in the mesenchyme of the cochlear and semicircular ducts. The expression of Cx37 in *yotari* mice was low, particularly in the epithelium of both the cochlear and semicircular ducts. However, it was lower than in wt mice at the same stages, except for the mesenchyme of the cochlear and semicircular ducts at E15.5, where Cx37 expression was significantly lower. Interestingly, the Cx37 expression in the human inner-ear developmental stages was significantly higher than in the corresponding wt and *yotari* mouse stages, except in the mesenchyme of the cochlear duct in the sixth week of development (Figure 4).

**Figure 5:**
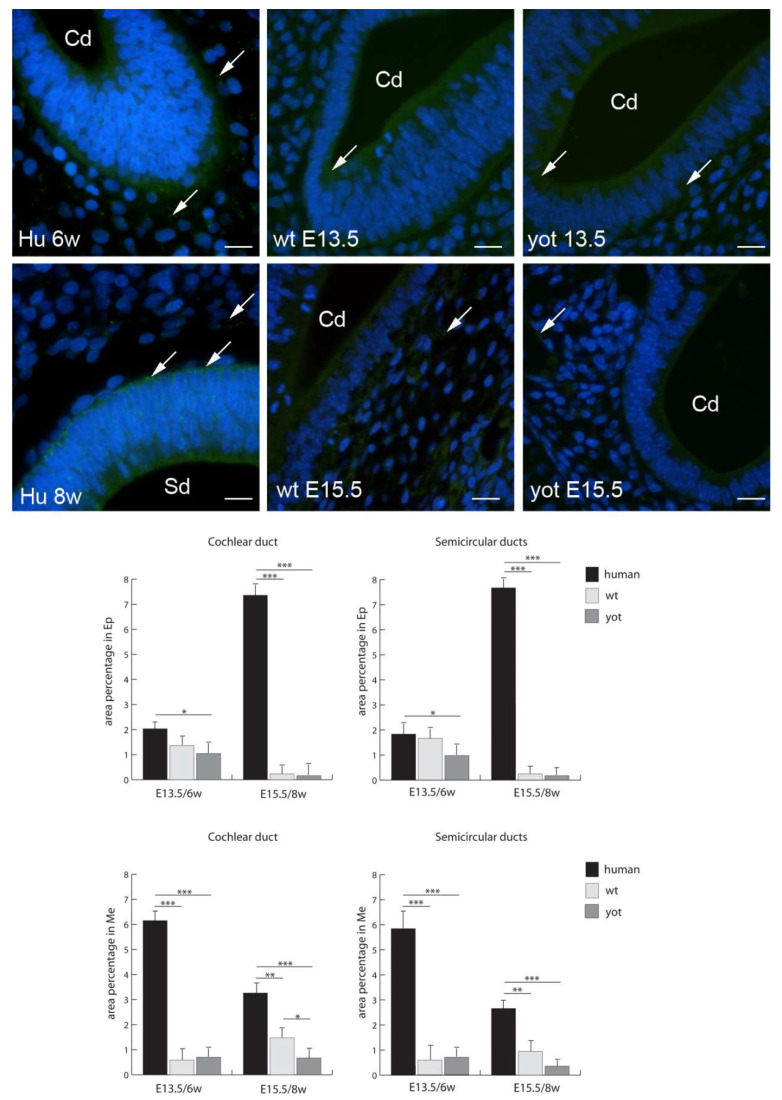
Immunofluorescence staining of connexin Cx40 (arrows) in the human (Hu), and wild-type (wt) and *yotari* (yot) mouse inner ears in the 6th and 8th weeks of human development and at corresponding E13.5 and E15.5, merged with DAPI (blue nuclear stain). Scale bar is 10 µm, for all images. The panel with graphs represents the area percentages of connexin Cx40 in the human, and wild-type and yotari mouse inner ears in the 6th and 8th weeks of human development and at corresponding E13.5 and E15.5 in epithelium and mesenchyme of cochlear duct and semicircular ducts. Sd (semicircular ducts), Cd (cochlear duct), Ep (epithelium), and Me (mesenchyme). Data are presented as the mean ± SD (vertical line). Significant differences are indicated by * *p* < 0.05, ** *p* < 0.01, and *** *p* < 0.001. One-way ANOVA followed by Tukey’s multiple-comparisons test.

Cx40 had the highest expression in the epithelium and mesenchyme of the cochlear and semicircular ducts in the human inner ear in both the sixth and eighth weeks of development and was statistically higher than in *yotari* mice for all the corresponding structures and stages. Similarly, Cx40 was always higher in the human inner ear than in wt mice, but with statistical significance only in the epithelium of the semicircular duct in the eighth week of development and in the mesenchyme for all the structures and stages (Figure 5). The lowest expression of Cx40 was in both the epithelium and mesenchyme of all the stages and inner-ear structures of *yotari* mice except in the mesenchyme of the cochlear and semicircular ducts at E13.5, where it was insignificantly lower than that in wt mice. From the sixth to the eighth week of human inner-ear development, there was a strong increase in Cx40 expression in the epithelium of the cochlear and semicircular ducts, while in the mesenchyme, there was a strong decrease in Cx40 expression in the cochlear and semicircular ducts. Contrary to this, the Cx40 pattern in the humans and wt mice displayed the opposite trend, with a decrease in the epithelium and increase in the mesenchyme of the cochlear and semicircular ducts (Figure 5).

**Figure 6:**
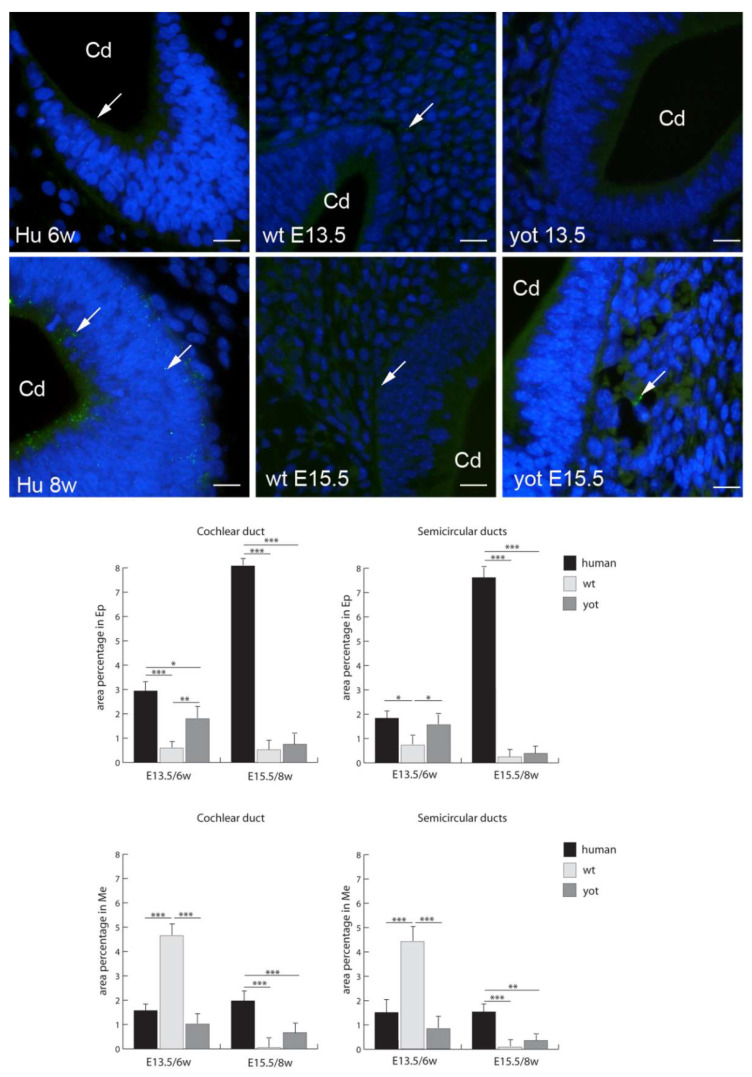
Immunofluorescence staining of connexin Cx43 (arrows) in the human (Hu), and wild-type (wt) and *yotari* (yot) mouse inner ears in the 6th and 8th weeks of human development and at corresponding E13.5 and E15.5, merged with DAPI (blue nuclear stain). Scale bar is 10 µm, for all images. The panel with graphs represents the area percentages of connexin Cx43 in the human, and wild-type and yotari mouse inner ears in the 6th and 8th weeks of human development and at corresponding E13.5 and E15.5 in epithelium and mesenchyme of cochlear duct and semicircular ducts. Sd (semicircular ducts), Cd (cochlear duct), Ep (epithelium), and Me (mesenchyme). Data are presented as the mean ± SD (vertical line). Significant differences are indicated by * *p* < 0.05, ** *p* < 0.01, and *** *p* < 0.001. One-way ANOVA followed by Tukey’s multiple-comparisons test.

Cx43 had the highest expression in the epithelium of the cochlear and semicircular ducts in the human inner ear in the eighth week of development, while in the mesenchyme, Cx43 expression was the highest in the cochlear and semicircular ducts of E13.5 wt mice (Figure 6). The expression of Cx43 was statistically higher than in *yotari* mice for all the corresponding structures and stages, except in the epithelium of the semicircular ducts and mesenchyme of the cochlear and semicircular ducts in the sixth week of development. Additionally, Cx43 was statistically higher in the human inner ear than in wt mice in all the stages and structures, except in the mesenchyme of the cochlear and semicircular ducts at E13.5, where Cx43 was statistically higher in the wt mice. The lowest expression of Cx43 was in the mesenchyme of the cochlear and semicircular ducts of wt mice at E15.5 (Figure 6).

**Figure 7:**
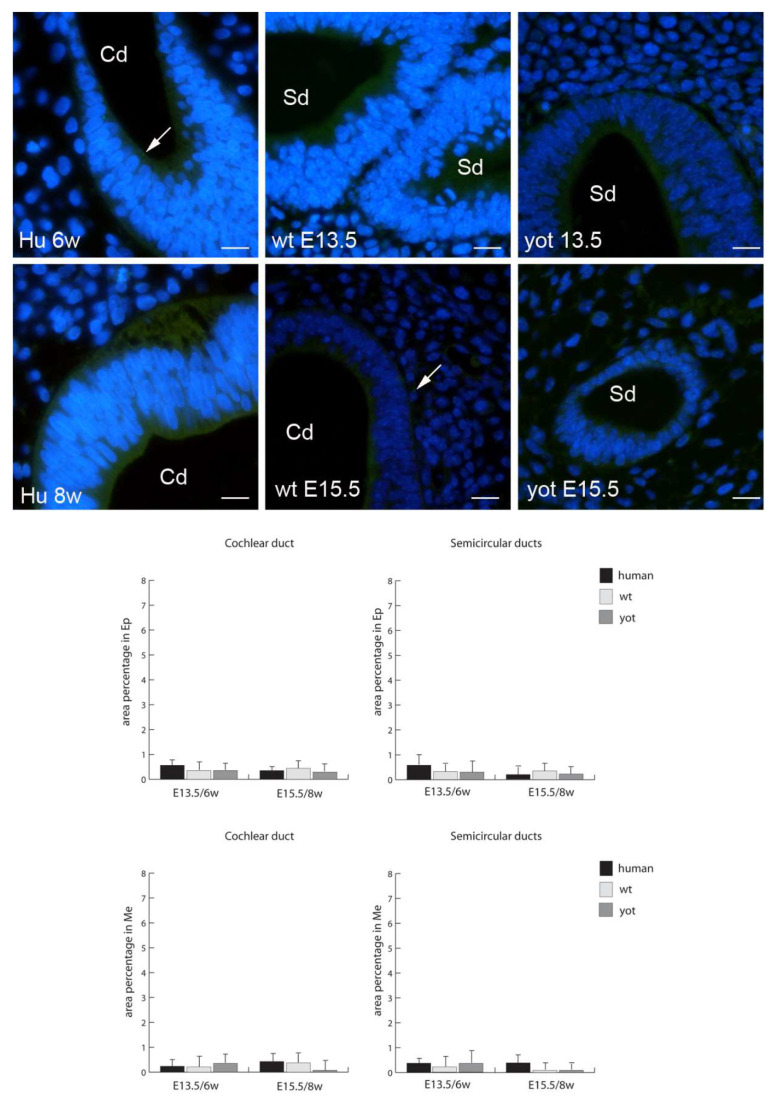
Immunofluorescence staining of connexin Cx45 (arrows) in the human (Hu), and wild-type (wt) and *yotari* (yot) mouse inner ears in the 6th and 8th weeks of human development and at corresponding E13.5 and E15.5, merged with DAPI (blue nuclear stain). Scale bar is 10 µm, for all images. The panel with graphs represents the area percentages of connexin Cx45 in human, and wild-type and yotari mouse inner ears in the 6th and 8th weeks of human development and at corresponding E13.5 and E15.5 in epithelium and mesenchyme of cochlear duct and semicircular ducts. Sd (semicircular ducts), Cd (cochlear duct), Ep (epithelium), and Me (mesenchyme). Data are presented as the mean ± SD (vertical line). There were no significant differences according to one-way ANOVA and Tukey’s multiple-comparisons tests.

The expression of Cx45 was almost non-existent in all the humans, and wt and yotari mice, in all the structure and stages, without statistical significance (Figure 7).

**Figure 8:**
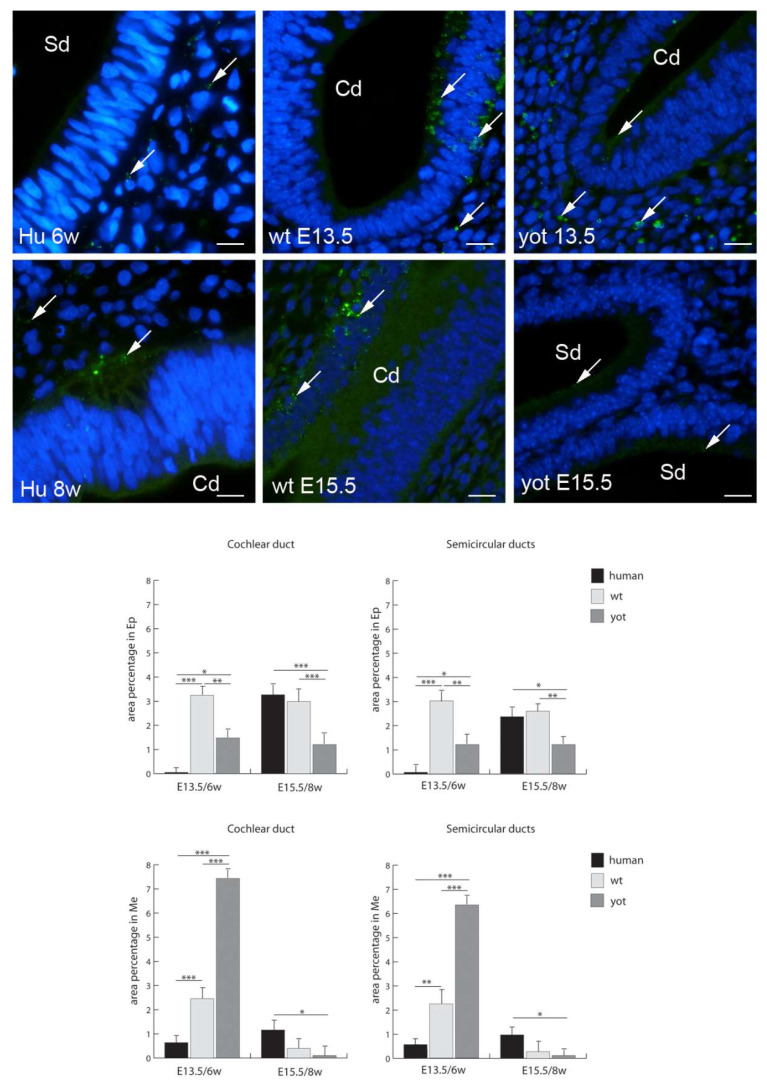
Immunofluorescence staining of pannexin 1 (Panx1) (arrows) in the human (Hu), and wild-type (wt) and *yotari* (yot) mouse inner ears in the 6th and 8th weeks of human development and at corresponding E13.5 and E15.5, merged with DAPI (blue nuclear stain). Scale bar is 10 µm, for all images. The panel with graphs represents the area percentages of Panx1 in the human, and wild-type and yotari mouse inner ears in the 6th and 8th weeks of human development and at corresponding E13.5 and E15.5 in epithelium and mesenchyme of cochlear duct and semicircular ducts. Sd (semicircular ducts), Cd (cochlear duct), Ep (epithelium), and Me (mesenchyme). Data are presented as the mean ± SD (vertical line). Significant differences are indicated by * *p* < 0.05, ** *p* < 0.01, and *** *p* < 0.001. One-way ANOVA followed by Tukey’s multiple-comparisons test.

Panx1 had the highest expression in the mesenchyme of the cochlear and semicircular ducts in the *yotari* inner ear at E13.5, and at the same stage, the human inner ear had the lowest Panx1 expression in the epithelium (Figure 8). From the sixth to the eighth week of human inner-ear development, there was a strong increase in Panx1 expression in the epithelium of the cochlear and semicircular ducts, while in the mesenchyme, there was a mild increase in Panx1 expression in these structures. The expression of Panx1 was significantly lower in the epithelium of the cochlear and semicircular ducts of *yotari* mice in comparison to wt mice and humans except in the epithelium of the cochlear and semicircular ducts and the mesenchyme of the cochlear duct of humans in the sixth developmental week. Contrary to that, the Panx1 expression in *yotari* mice was significantly higher in the mesenchyme of the cochlear and semicircular ducts at E13.5 in comparison to humans and wt mice (Figure 8).

## 4. Discussion

Although yotari mice are mostly investigated for brain development, studies about their inner-ear development are lacking. It is known that yotari mice have disturbances in layer 5 of the inferior colliculi cortex, which is a hearing pathway [22], and that reelin is involved in cases of adult otosclerosis due to the coordinative role of reelin in the early embryonic stages of development [23]. The connection between the reelin signalling pathway and Cxs and Panx1, and their possible role in cochlear or vestibular impairment have not been investigated till now. However, normal inner-ear development is highly dependent on gap junctional intercellular communication and hemichannel formation, because they are crucial for the passive intercellular diffusion of small hydrophilic molecules [10]. Although the complete mechanism of Cxs’ impact on inner-ear function is still in dispute, it has been recognized that Cxs might have an important role in inner-ear networking since their mutations, especially of Cx26, are involved in syndromic and non-syndromic deafness or loss of saccular function [24,25]. Cx26 is diminished in the cochleae of patients with otosclerosis and contributes to the hyalinization of the spiral ligament and loss of type II and type III fibrocytes [26].

In order to elucidate Cxs’ and Panx1’s involvement in inner-ear development at the embryonic stages E13.5 and E15.5 (the sixth and eighth weeks of development in humans, respectively), we separately investigated Cx and Panx1 expression in the epithelium and mesenchyme. Namely, epithelium and mesenchyme tissues provide the epithelial and the connective tissue gap junctional network in the inner ear that starts to develop from E16 and around postnatal P0, respectively [10].

In the investigated developmental periods, Cxs and Panx1 showed different expression patterns in the epithelial vs. connective tissues, also providing some conflicting results in the comparison of humans and wt mice with the yotari development. Namely, the majority of the examined Cxs were significantly downregulated in the cochlear and the semicircular ducts of yotari mice compared to wt mice and humans, except for Cx32, which was significantly higher in yotari. These results might imply a compensatory increase in Cx32 in yotari as a response to the downregulation of other investigated Cxs. This conclusion is in reasonable agreement with a report that showed that the ablation of Cx26 in the cochlea was associated with the downregulation of Cx30 [2]. Additionally, Degen et al. reported that Cx32 could functionally replace Cx26 in Cx26-deficient mice, and that such mice had almost no hearing impairment [7].

Cx26 and Cx32 are known to be expressed in the epithelia and connective tissue components of the cochlea, and they establish a functional syncytium between them [27]. In the mature inner ear, Cx26 and Cx32 are presented between the adjacent supporting cells in both the vestibular and auditory sensory epithelia [28]. In a common marmoset, Cx26 at E96 was expressed in the prosensory domain, in a part of the modiolus and lateral sides of the sensory epithelium, but later, at E101, Cx26 expression was still observed in the sensory epithelium, except in the organ of Corti, while at E115, weak expression was observed in the spiral ligament fibrocytes next to the stria [29].

In our study, the expression of Cx26 in the epithelium of the cochlear and semicircular ducts was three- to four-fold lower in yotari than in wt mice. However, in both the epithelium and mesenchyme of the cochlear duct at the E15.5 stage, the expression of Cx32 was significantly higher than in wt mice. This might imply that Cx32 can compensate for a low expression level of Cx26 [30]. At the same time, the expression of Cx32 in the human inner ear during the sixth and eighth developmental weeks was very low, which might indicate that this particular connexin is not paramount in these periods for human inner-ear development.

In our study, high expression of Cx26, Cx40, and Cx43 was observed in the epithelium during human inner-ear development. In wt mice, high expression of Cx40 was observed in the epithelium, while Cx43 dominated in the mesenchyme. The expression of Cx26, Cx40, and Cx43 was significantly lower in *yotari*. These findings might highlight the importance of the proper expression of these Cxs in gap junction intercellular communication (GJIC) to provide avascular sensory epithelium with key metabolites and glucose, but also K+ recycling [8]. On the other hand, Cx26 expression dominated in the mesenchyme of wt and yotari, while Cx37 dominated in the mesenchyme of the cochlear and semicircular ducts in both human and mouse embryos. The latter finding might be associated with vascular development, since Cx37 is involved in different vascular pathologies [31]. Mice with Cx37 dab1−/− showed problems with body water handling via the kidneys, resulting in polyuria and polydipsia [32]. In addition, Cx40 displayed a more specific pattern in human samples and was expressed mostly in the capillary network. Their roles might be important in the pericytes of the blood vessels of the spiral ligament, where they have been found across different types of microvessels [33].

In our study, Cx32 was more intensely expressed in mouse inner-ear development, while Cx26, Cx37, Cx43, and Panx1 seem to be relevant for both human and mouse embryos. The expression of Cx26, Cx37, and Cx43 seems to be overlapping in the organ of Corti [34], while in advanced stages of cochlear development, Cx43 can be observed only in the region of tension fibrocytes lining the inner aspect of the otic capsule [28]. Using the lacZ reporter gene in Cx43 KO mice, Cohen-Salmon et al. revealed that Cx43 was highly expressed in the connective tissues and weakly expressed in the immature sensory epithelium of the cochlea from embryonic day 15.5 [13]. In our study, the expression of Cx43 in the epithelium and mesenchyme at E15.5 did not significantly differ between wt and yotari mice, and the expression was weak. However, the Cx43 expression in the human inner ear at the same stages was significantly higher, thus indicating the relevance of Cx43 in human inner-ear development. In addition, it was shown that Cx43 may be associated with the development of middle ear cholesteatoma in humans [35]. In our study, Cx45 had negligible expression in both mice and human embryos. This finding is in line with a study that used in situ hybridization, providing information showing low expression of Cx45 in the organ of Corti [34]. In addition, Cohen-Salmon et al. found the expression of Cx45 in the mouse inner ear from E15.5 onward and in the mature inner ear, where Cx45 was expressed in the entire vasculature [13]. These results indicate that Cx43 and Cx45 might play a role in the development of the otic capsule bone and the inner-ear vascular system.

We used Panx1 expression to assume potential channel formation. At E13.5, we observed a strong increase in the expression of Panx1 in the mesenchyme of yotari compared to wt. However, yotari mice had significantly lower Panx1 expression in the epithelium of the cochlear and semicircular ducts than the inner ears of wt and human embryos. This finding might be in line with a role for Panx1 hemichannels in the propagation of intercellular calcium ion waves in the developing inner ear [10].

## 5. Conclusions

In conclusion, our data could provide important information for better understanding disorders that can arise during inner-ear development, since Cxs and Panx1 seem to be key players in the maintenance of inner-ear homeostasis. Therefore, Cxs and Panx1 could serve as potential therapeutical targets in inner-ear pathology and diagnostics. Moreover, we emphasize the involvement of Cxs and Panx during early inner-ear development and possible involvement of the reelin signalling pathway. Further studies are required to elucidate whether a network between reelin signalling and GJIC has a role in hearing and balance impairment. Considering that the reelin signalling pathway is active in adult otosclerosis and inferior colliculi impairments, it could affect transport through GJ in inner-ear embryogenesis as well.

## Figures and Tables

**Table 1:** List of primary and secondary antibodies.

Antibodies		Host	Dilution	Source	Catalogue No.
Primary	Cx26, GJB2	Rabbit	1:50	Cusabio (Wuhan, China)	CSBPA009452LA01HU
	Cx32, GJB1	Rabbit	1:100	Cusabio (Wuhan, China)	CSB-PA008853
	Anti-Cx37/GJA4	Rabbit	1:300	Abcam (Cambridge, UK)	ab181701
	Anti-Cx40/GJA5	Rabbit	1:50	Abcam (Cambridge, UK)	ab213688
	Anti-Cx43/GJA1	Goat	1:100	Abcam (Cambridge, UK)	ab87645
	Anti-Cx45/GJA7	Rabbit	1:50	Abcam (Cambridge, UK)	ab135474
	Anti-pannexin 1/PANX1	Rabbit	1:150	Merck KGaA (Darmstadt, Germany)	ABN242
Secondary	Donkey Anti-Goat IgG Alexa Fluor 488	Donkey	1:400	Abcam (Cambridge, UK)	ab150129
	Donkey Anti-Rabbit IgG Alexa Fluor 488	Donkey	1:400	Abcam (Cambridge, UK)	ab150073

## Data Availability

The data presented in this study are available on request from the corresponding author.
